# 11,12-EET Stimulates the Association of BK Channel α and β_1_ Subunits in Mitochondria to Induce Pulmonary Vasoconstriction

**DOI:** 10.1371/journal.pone.0046065

**Published:** 2012-09-24

**Authors:** Annemarieke E. Loot, Isabelle Moneke, Benjamin Keserü, Matthias Oelze, Tetyana Syzonenko, Andreas Daiber, Ingrid Fleming

**Affiliations:** 1 Institute for Vascular Signalling, Centre for Molecular Medicine, Goethe University, Frankfurt am Main, Germany; 2 2^nd^ Medical Clinic, Department of Cardiology, Johannes Gutenberg University, Mainz, Germany; University of Giessen Lung Center, Germany

## Abstract

In the systemic circulation, 11,12-epoxyeicosatrienoic acid (11,12-EET) elicits nitric oxide (NO)- and prostacyclin-independent vascular relaxation, partially through the activation of large conductance Ca^2+^-activated potassium (BK) channels. However, in the lung 11,12-EET contributes to hypoxia-induced pulmonary vasoconstriction. Since pulmonary artery smooth muscle cells also express BK channels, we assessed the consequences of BKβ_1_ subunit deletion on pulmonary responsiveness to 11,12-EET as well as to acute hypoxia. In buffer-perfused mouse lungs, hypoxia increased pulmonary artery pressure and this was significantly enhanced in the presence of NO synthase (NOS) and cyclooxygenase (COX) inhibitors. Under these conditions the elevation of tissue EET levels using an inhibitor of the soluble epoxide hydrolase (sEH-I), further increased the hypoxic contraction. Direct administration of 11,12-EET also increased pulmonary artery pressure, and both the sEH-I and 11,12-EET effects were prevented by iberiotoxin and absent in BKβ_1_^−/−^ mice. In pulmonary artery smooth muscle cells treated with NOS and COX inhibitors and loaded with the potentiometric dye, di-8-ANEPPS, 11,12-EET induced depolarization while the BK channel opener NS1619 elicited hyperpolarization indicating there was no effect of the EET on classical plasma membrane BK channels. In pulmonary artery smooth muscle cells a subpopulation of BK channels is localized in mitochondria. In these cells, 11,12-EET elicited an iberiotoxin-sensitive loss of mitochondrial membrane potential (JC-1 fluorescence) leading to plasma membrane depolarization, an effect not observed in BKβ_1_^−/−^ cells. Mechanistically, stimulation with 11,12-EET time-dependently induced the association of the BK α and β_1_ subunits. Our data indicate that in the absence of NO and prostacyclin 11,12-EET contributes to pulmonary vasoconstriction by stimulating the association of the α and β_1_ subunits of mitochondrial BK channels. The 11,12-EET-induced activation of BK channels results in loss of the mitochondrial membrane potential and depolarization of the pulmonary artery smooth muscle cells.

## Introduction

Acute hypoxic pulmonary vasoconstriction is an adaptive response of the pulmonary circulation that directs blood flow from poorly oxygenated to better ventilated areas thereby maintaining pulmonary gas exchange [1]. To-date, the pulmonary oxygen sensor and the signaling cascade leading to hypoxic pulmonary vasoconstriction have not been fully elucidated. The most likely candidates for pulmonary O_2_ sensing are the mitochondria of the pulmonary resistance artery smooth muscle cells. Although it is unclear whether mitochondria increase or decrease reactive oxygen species (ROS) output in response to hypoxia, inhibition of the electron transport chain, as occurs under moderate hypoxia, does seem to be a prerequisite for hypoxic pulmonary vasoconstriction [2], [3]. Eventually, the changes in redox status combined with other intracellular mediators activate the ion channels that elicit the changes in intracellular Ca^2+^ and Rho kinase activity that ultimately lead to vasoconstriction [4], [5]. Among the intracellular mediators that are generated during acute hypoxia are the cytochrome P450 (CYP)-derived epoxyeicosatrienoic acids (EETs) [6], [7], and preventing their metabolism by inhibiting the soluble epoxide hydrolase (sEH) can markedly potentiate hypoxic pulmonary vasoconstriction [8].

How CYP epoxygenase-derived EETs regulate vascular tone has long been a subject of interest, but the molecular mechanisms involved a clear have yet to be completely clarified. In the systemic circulation, EET production is associated with nitric oxide (NO)- and prostacyclin (PGI_2_)-independent vasodilatation and a significant percentage of the acute vascular actions of EETs have been attributed to the activation of small and intermediate conductance Ca^2+^-sensitive K^+^ channels within endothelial cells or large conductance Ca^2+^-sensitive K^+^ (BK) channels (K_Ca_1.1, Slo, Slo1 [9]) in smooth muscle cells [10]. In the pulmonary circulation, EETs (in particular 11,12-EET) tend to do the opposite and stimulate vasoconstriction and an increase in pulmonary perfusion pressure [11]. Indeed, we recently reported that CYP epoxygenases are involved in the pulmonary vasoconstriction induced by acute exposure to hypoxia via a mechanism involving the intracellular translocation and activation of transient receptor potential (TRP) C6 channels [8]. CYP metabolites are also implicated in responses to chronic hypoxia as the pulmonary vascular remodeling that occurs during prolonged exposure to hypoxia is related to both the upregulation of CYP epoxygenase expression [6] and an almost complete loss of the sEH [12], effects that markedly increase tissue EET levels.

As our previous data demonstrated the presence of a functional CYP/sEH pathway in pulmonary vascular smooth muscle cells, the aim of the present investigation was to determine the role of the BK channel in hypoxia-induced pulmonary vasoconstriction. One mechanism regulating BK channel function is the association of the pore-forming α subunit with regulatory β subunits. Of the four BKβ subunit isoforms, the β1 subunit seems to be restricted to vascular smooth muscle cells [13], therefore we compared responses to 11,12-EET in wild-type and BKβ1 subunit-deficient (BKβ1^−/−^) mice.

## Methods

### Chemicals

The sEH inhibitor 1-adamantyl-3-cyclohexylurea (ACU) [14] was kindly provided by Bruce D. Hammock (UC Davis, California, USA) and the EET antagonist 14,15-epoxyeicosa-5(*Z*)-enoic acid (14,15-EEZE) [15] was from John R. Falck (University of Texas Southwestern Medical Center, Texas, USA). 11,12-EET was obtained from Cayman Chemicals (Massy, France), U46619 and JC-1 were from Alexis (Lörrach, Germany) di-8-ANEPPS from Invitrogen (Carlsbad, USA), and Rp-cAMPS from BioLog Life Science Institute (Bremen, Germany). N^ω^-nitro-L-arginine (L-NA) and all other substances were purchased from Sigma (Deisenhofen, Germany).

### Animals

BKβ1^−/−^ mice, generated as described [16], were bred by the animal facility at the University of Mainz and C57BL/6 mice (6–8 weeks old) were purchased from Charles River (Sulzfeld, Germany). Mice were housed in conditions that conform to the Guide for the Care and Use of Laboratory Animals published by the U.S. National Institutes of Health (NIH publication no. 85–23) and Directive 2010/63/EU of the European Parliament. Both the university animal care committee and the federal authority for animal research (Regierungspräsidium Darmstadt, Hessen, Germany) approved the study protocols (# F28/14).

### Isolated Buffer-perfused Mouse Lung

Changes in pulmonary perfusion pressure were assessed in the isolated buffer-perfused mouse lung, as described [17]. Briefly, mice were anaesthetized by intraperitoneal injection of ketamine and xylazine (80 mg/kg and 10 mg/kg, respectively), catheters were inserted into the pulmonary artery and left atrium and buffer perfusion via the pulmonary artery was initiated at a flow of 0.2 ml/min. Ventilation was then changed from room air to a pre-mixed gas (21% O_2_, 5% CO_2_, balanced with N_2_), left atrial pressure was set to 2.0 mmHg and flow was slowly increased from 0.2 to 2 mL/min. For hypoxic ventilation, a gas mixture containing 1% O_2_, 5% CO_2_, balanced with N_2_ was used. Ten minute periods of hypoxic ventilation were alternated with 15 minutes of normoxia.

### Cell Culture

Wild-type and BKβ1^−/−^ mice were euthanized in a closed chamber containing isoflurane-soaked gauzes followed by thoracotomy. Pulmonary artery smooth muscle cells were isolated as described [18] and cultured in M199, supplemented with 20% FCS, penicillin (50 U/mL) and streptomycin (50 µg/mL).

HEK293 cells were purchased from ATCC and transfected with plasmids encoding the flag-tagged BKα subunit (pM2AH-hSlo1 [19], addgene, Cambridge, MA) and/or the BKβ1 subunit (pCR3-hMaxiK β1; kindly provided by Annelies Janssens, KU Leuven, Leuven Belgium) using Lipofectamin™ (Invitrogen, Darmstadt, Germany). One day after transfection, the cells were placed in serum free medium and stimulated as indicated in the Results section.

### Cell Fractionation, Immunoprecipitation and Immunoblotting

To obtain mitochondrial membranes, HEK293 cells were harvested by scraping in ice cold PBS and re-suspended in hypotonic buffer (sucrose 83 mmol/L, Tris 6.7 mmol/L, pH 7.4) containing protease inhibitors. After fractionating the cells by repeated passage through a 30G needle and removal of nuclei and unbroken cells (600 *g*, 10 minutes) the mitochondria and mitochondrial membranes were recovered by centrifugation at 10,000 *g* (10 minutes).

For immunoprecipitation, HEK293 cells were lysed in Nonidet buffer. The lysate was cleared by centrifugation at 1,000 *g* and the BKβ1 subunit was immunoprecipitated with specific antibodies (sc-14749, Santa Cruz Biotechnology, Heidelberg, Germany). Mouse pulmonary artery smooth muscle cells were solubilized in SDS sample buffer. Cell lysates, mitochondrial membranes, and immunoprecipitates were separated by SDS-PAGE, and transferred to a nitrocellulose membrane and probed with specific antibodies against the BKβ1 subunit (Santa cruz), the flag-tagged BKα subunit (Sigma), β-actin (Sigma), and the voltage-dependent anion-selective channel 1 (VDAC1, Abcam) as described [20].

### Measurement of Pulmonary Artery Smooth Muscle Cell Membrane Potentials

Changes in plasma membrane potential were assessed using the potentiometric fluorescent dye di-8-ANEPPS (excitation λ475 nm and emission λ550 and 620 nm) using a spectrofluorimeter, as described [21]. Briefly, pulmonary artery smooth muscle cells cultured on glass coverslips were incubated with di-8-ANEPPS (2 µmol/L) and 0.5% Pluronic F127 (Molecular Probes, Leiden, the Netherlands) for 20 minutes, washed 3 times and incubated for 20 minutes in HEPES-buffered Tyrode’s solution containing L-NA and diclofenac before data were collected. An increase in the λ550/620 emission ratio indicates membrane depolarization. Results were normalized to the membrane depolarization elicited by 80 mmol/L extracellular K^+^.

To assess changes in mitochondrial membrane potential (ΔΨ_m_), pulmonary artery smooth muscle cells were loaded with JC-1 (5 µmol/L) for 15 minutes and measurements were performed as above (excitation λ490 nm, emission λ530 and 590 nm). An increase in the 530/590 emission ratio indicates a decrease in the ΔΨ_m_. Results were normalized to the effect of the mitochondrial uncoupler carbonyl cyanide 3-chlorophenylhydrazone (CCCP, 1 µmol/L).

### Isolation of Mitochondria from the Mouse Lung

Lung mitochondria were isolated as described [22]. Briefly, lungs were minced and homogenized in isolation buffer (250 mmol/L sucrose, 2 mmol/L EDTA, 5 mmol/L Tris HCl pH 7.4 and 0.5% BSA) in a glass Potter- Elvehjem homogenizer with a Teflon pestle. The lysate was cleared by 5 minutes centrifugation at 2000 g, 4°C, and mitochondria were pelleted at 10,000 g, 5 minutes, 4°C. The mitochondria were further purified by repeated resuspension and centrifugation in BSA-free isolation buffer followed by density gradient centrifugation. Mitochondrial membrane potential was measured as described [23], in a buffer containing 110 mmol/L KCl, 0.5 mmol/L KH_2_PO_4_, 1 mmol/L MgCl_2_, 10 µmol/L EGTA, 10 mmol/L succinate, 2 mmol/L malate, 20 mmol/L K-HEPES, pH 7.2, and 2 µmol/L JC-1.

### Statistical Analysis

Data are expressed as the mean ± SEM and statistical evaluation was performed using either Student’s t test for unpaired data or 2-way ANOVA followed by Bonferroni’s t test where appropriate. Values of P<0.05 were considered statistically significant.

## Results

### Contribution of the BKβ1 Subunit to Acute Hypoxic Vasoconstriction and Sensitivity to sEH Inhibition

**Figure 1:**
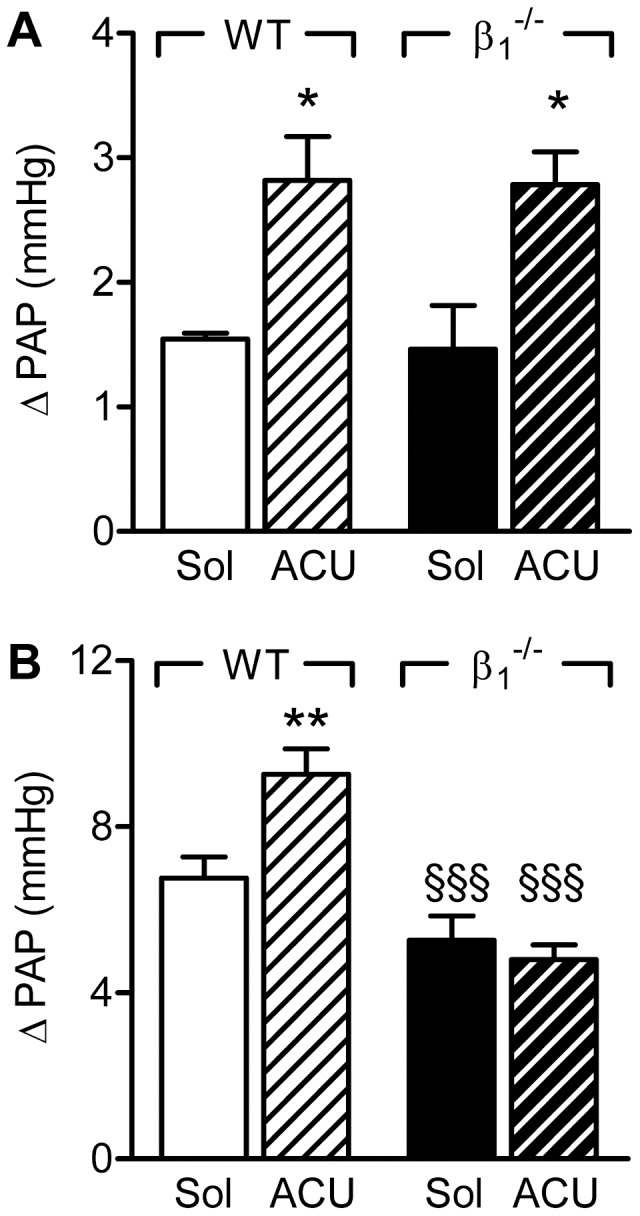
Effect of sEH inhibition on hypoxic pulmonary vasoconstriction in wild-type and BKβ_1_^−/−^ mice. Hypoxia-induced increases in pulmonary arterial pressure (ΔPAP) were assessed in the presence of solvent (Sol) or ACU (3 µmol/L) in isolated lungs from wild-type (WT) or BKβ_1_^−/−^ mice. Experiments were performed in (A) the absence and (B) the presence of diclofenac (10 µmol/L) and L-NA (300 µmol/L). The graphs summarize data obtained in 4–15 independent experiments; *P<0.05, **P<0.01 versus Sol; §§§P<0.001 versus WT+ACU.

Hypoxic ventilation (F_i_O_2_ = 0.01) of lungs from wild-type mice resulted in an acute increase in pulmonary artery pressure and a similar response was observed using lungs from BKβ1^−/−^ mice (Figure 1A). Following inhibition of the sEH with ACU to increase pulmonary EET accumulation, the acute hypoxic vasoconstriction was significantly enhanced in both mouse strains (Figure 1A).

As vascular responses that are dependent on the activation of BK channels are frequently only detectable in the absence of NO and PGI_2_ [24], we assessed the sensitivity of acute hypoxic vasoconstriction to sEH inhibition in the presence of L-NA (300 µmol/L) and diclofenac (10 µmol/L). In lungs from wild-type animals, combined NO synthase (NOS) and cyclooxygenase (COX) inhibition significantly increased the magnitude of the acute hypoxic vasoconstriction (from 1.5±0.1 to 6.7±0.5 mmHg, n = 7–11, P<0.001), and this was increased even further by the sEH inhibitor (Figure 1B). The inhibition of NO and PGI_2_ also increased hypoxic vasoconstriction in lungs from BKβ1^−/−^ mice, albeit to a lesser extent than in the wild-type mice. However, under these conditions the addition of ACU to BKβ1^−/−^ lungs failed to potentiate hypoxic vasoconstriction.

**Figure 2:**
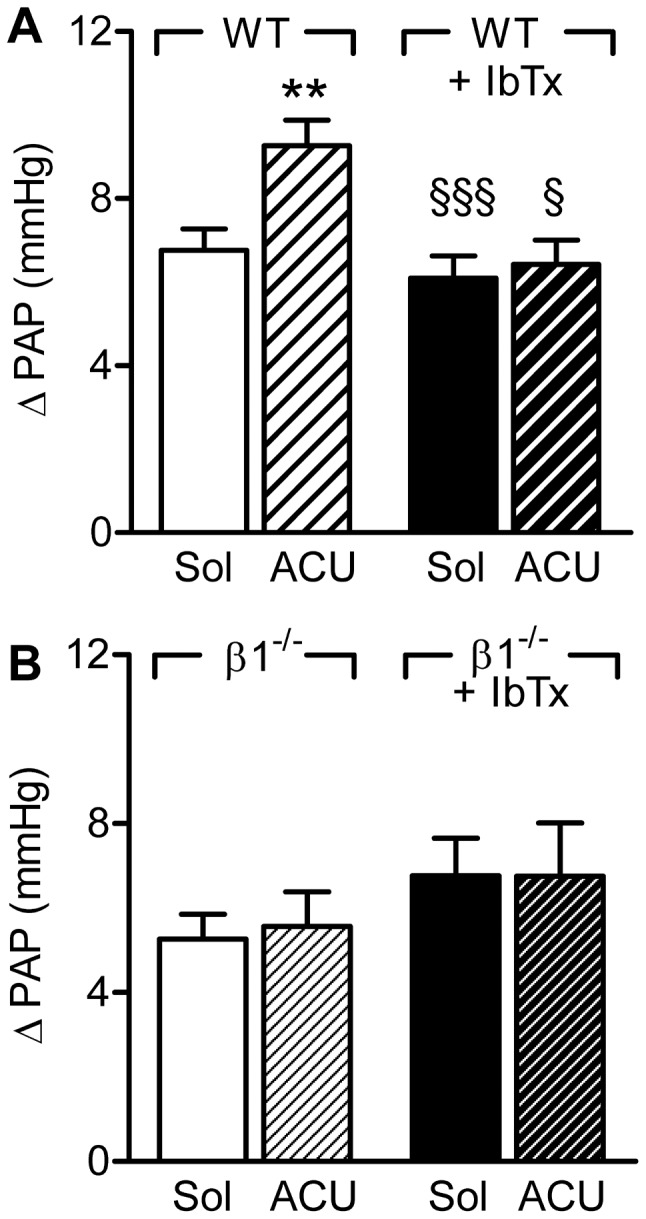
Effect of iberiotoxin on the sensitivity of the acute hypoxic vasoconstriction to sEH inhibition. Hypoxia-induced increases in pulmonary arterial pressure (ΔPAP) were assessed in the presence of solvent (Sol), ACU (3 µmol/L) and iberiotoxin (IbTx, 300 nmol/L) in isolated lungs from (A) wild-type (WT) or (B) BKβ_1_^−/−^ mice. All experiments were performed in the presence of diclofenac and L-NA. The graphs summarize data obtained in 7–13 independent experiments; **P<0.01 versus Sol; §P<0.05, §§P<0.001 versus WT+ACU.

We next assessed the effect of iberiotoxin (100 nmol/L) on the hypoxic vasoconstriction in lungs from wild-type and BKβ1^−/−^ mice in the combined presence of NOS and COX inhibitors. As before, hypoxic vasoconstriction in lungs from wild-type mice was increased by sEH inhibition (Figure 2A). Iberiotoxin did not affect basal acute hypoxic vasoconstriction but abrogated the effect of ACU. Hypoxic vasoconstriction was insensitive to sEH inhibition in lungs from BKβ1^−/−^ mice and iberiotoxin failed to modify these responses (Figure 2B). These findings indicate that the ACU-induced increase in pulmonary vasoconstriction in the absence of NO and PGI_2_, can be attributed to the activation of BK channels, via the BKβ1 subunit.

### Effect of Exogenously Applied 11,12-EET on Hypoxia-induced Vasoconstriction and Pulmonary Artery Pressure

**Figure 3:**
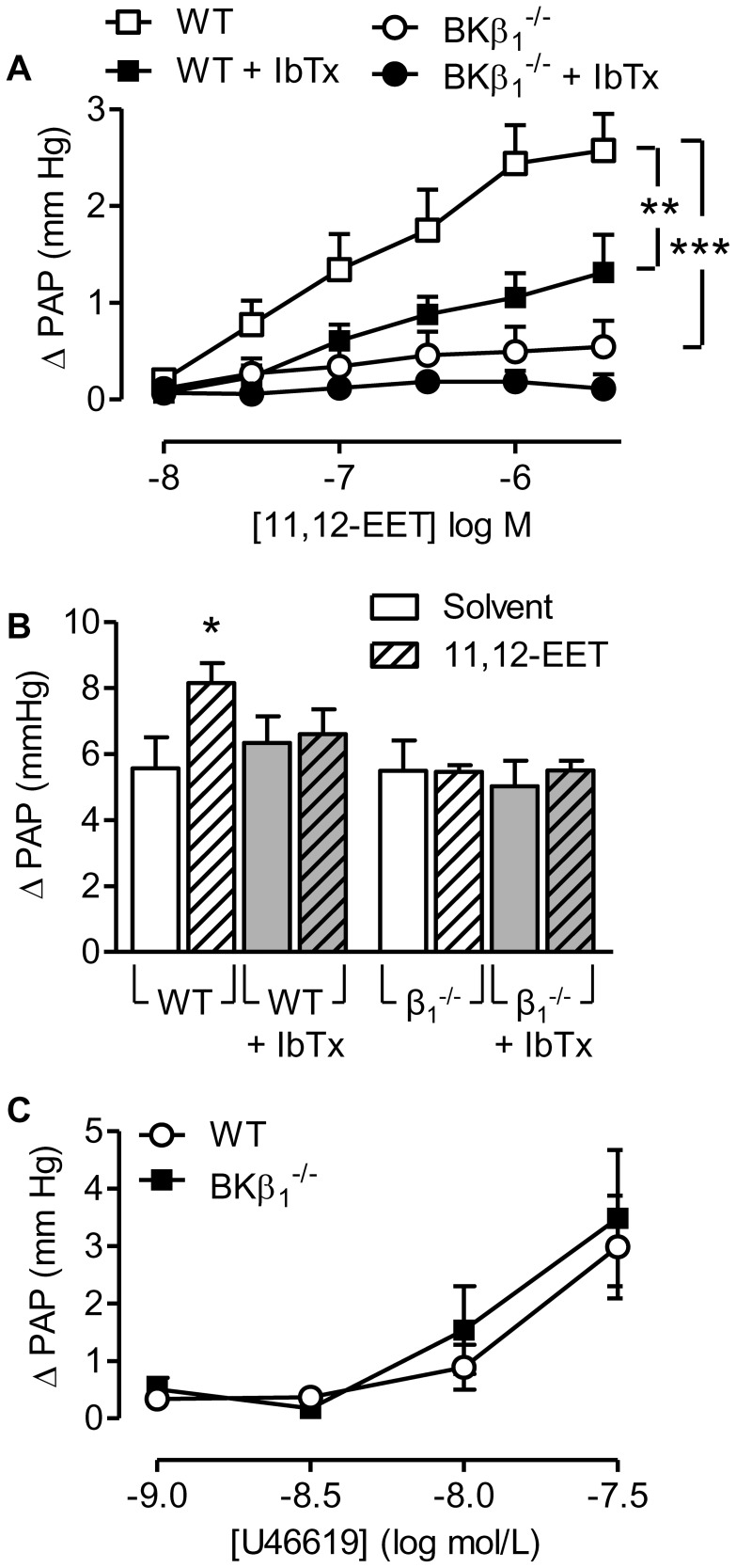
Effect of 11,12-EET on pulmonary artery pressure in lungs from wild-type (WT) and BKβ_1_^−/−^ mice. (A) 11,12-EET (10 nmol/L to 3 µmol/L)-induced increase in pulmonary arterial pressure (ΔPAP) in the absence or presence of iberiotoxin (IbTx, 300 nmol/L). (B) Effect of solvent, and 11,12-EET (3 µmol/L) on the hypoxia-induced increase in pulmonary arterial pressure (ΔPAP), in the absence and presence of IbTx. (C) Effect of U46619 (1–300 nmol/L) on pulmonary arterial pressure (ΔPAP) in lungs from WT and BKβ_1_^−/−^ mice. All experiments were performed in the presence of diclofenac (10 µmol/L) and L-NA (300 µmol/L). The graphs summarize data obtained in 4–8 independent experiments; *P<0.05, **P<0.01, ***P<0.001 versus WT.

To confirm that the wild-type and BKβ1^−/−^ mice respond differently to EETs, we analyzed the effect of 11,12-EET on pulmonary arterial pressure and its ability to potentiate acute hypoxic pulmonary vasoconstriction in the presence of NOS and COX inhibitors. In lungs from wild-type mice, 11,12-EET (10 nmol/L to 3 µmol/L) elicited a concentration-dependent increase in pulmonary artery pressure that was significantly attenuated in the presence of iberiotoxin (Figure 3A). However in lungs from BKβ1^−/−^ mice, the 11,12-EET-induced increase in pulmonary artery pressure was markedly attenuated with only a small residual response remaining and pre-incubation of these lungs with iberiotoxin was without significant effect (Figure 3A). Similarly, in lungs from wild-type mice, acute hypoxia elicited a vasoconstriction that was enhanced in the presence of 11,12-EET (Figure 3B). The latter effect was sensitive to the addition of iberiotoxin, again indicating dependence on the activation of BK channels, and was not observed in lungs from BKβ1^−/−^ mice (Figure 3B).

Under the same experimental conditions i.e. combined NOS/COX inhibition, the thromboxane mimetic U46619 elicited a potent vasoconstriction, that was identical in lungs from wild-type and BKβ1^−/−^ mice (Figure 3C, Figure S1). Thus, the deletion of the β1 subunit did not globally alter responsiveness to all pulmonary vasoconstrictors.

### Effect of 11,12-EET on Pulmonary Artery Smooth Muscle Cell Membrane Potential

**Figure 4:**
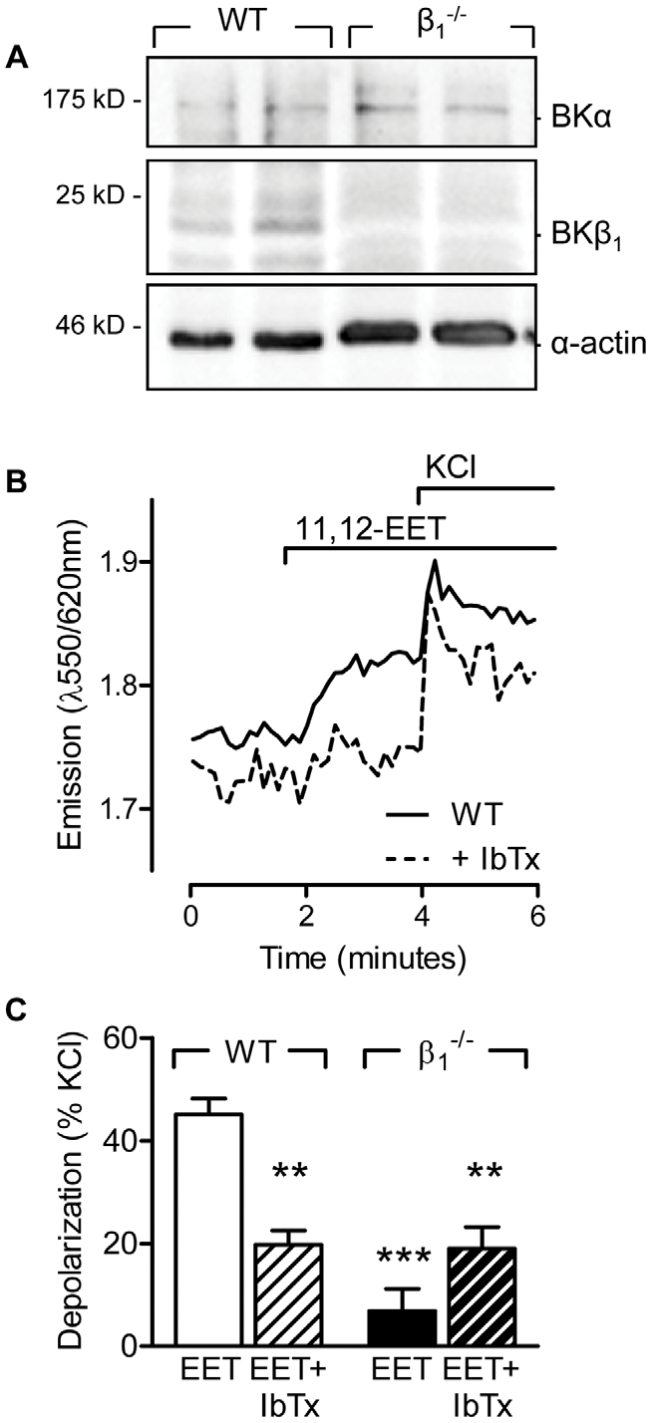
Effect of 11,12-EET on the membrane potential of wild-type and BKβ_1_^−/−^ pulmonary artery smooth muscle cells. (A) Representative blots showing the expression of the α and β_1_ subunits of the BK in cultured pulmonary artery smooth muscle cells. (B) Original tracing showing the effect of 11,12-EET (10 µmol/L) on the fluorescence emission ratio of Di-8-ANEPPS-loaded pulmonary artery smooth muscle cells. (C) Effect of 11,12-EET (10 µmol/L) on the membrane potential of wild-type (WT) and BKβ_1_^−/−^ pulmonary artery smooth muscle cells in the presence of solvent (Sol) or iberiotoxin (IbTx, 300 nmol/L). Experiments were performed in the presence of diclofenac (10 µmol/L) and L-NA (300 µmol/L). The bar graph summarizes data obtained in 4–8 independent experiments; **P<0.01, ***P<0.001 versus WT+Sol.

To determine the molecular mechanisms underlying the EET-induced vasoconstriction we isolated pulmonary artery smooth muscle cells from wild-type and BKβ1^−/−^ mice. We confirmed that the BKβ1 subunit was expressed in cultured pulmonary smooth muscle cells from wild-type animals up to passage 8, but was not detectable in cells from BKβ1^−/−^ mice (Figure 4A). To assess the consequences of 11,12-EET on membrane potential we loaded cultured murine pulmonary artery smooth muscle cells with the potential sensitive indicator di-8-ANEPPS. Depolarization of the cells with increasing concentrations of KCl resulted in an increase in the ANEPPS emission ratio (Figure S2A). The addition of the BK channel opener, NS1619 (1 µmol/L), caused a pronounced hyperpolarization of both wild-type and BKβ1^−/−^ cells (figure S2B) showing that the BKα subunit was present and functional in both cell types.

In wild-type pulmonary artery smooth muscle cells treated with L-NA and diclofenac, 11,12 EET (10 µmol/L) elicited smooth muscle cell depolarization and this effect was significantly diminished in the presence of iberiotoxin (Figure 4B and C). The 11,12 EET-induced membrane depolarization (but not that to KCl) was markedly reduced in pulmonary artery smooth muscle cells from BKβ1^−/−^ mice (Figure 4C). From these data we conclude that 11,12-EET does not activate the classical NS1619-sensitive BK channel population at the plasma membrane.

### Effect of 11,12-EET on ΔΨ_m_

**Figure 5:**
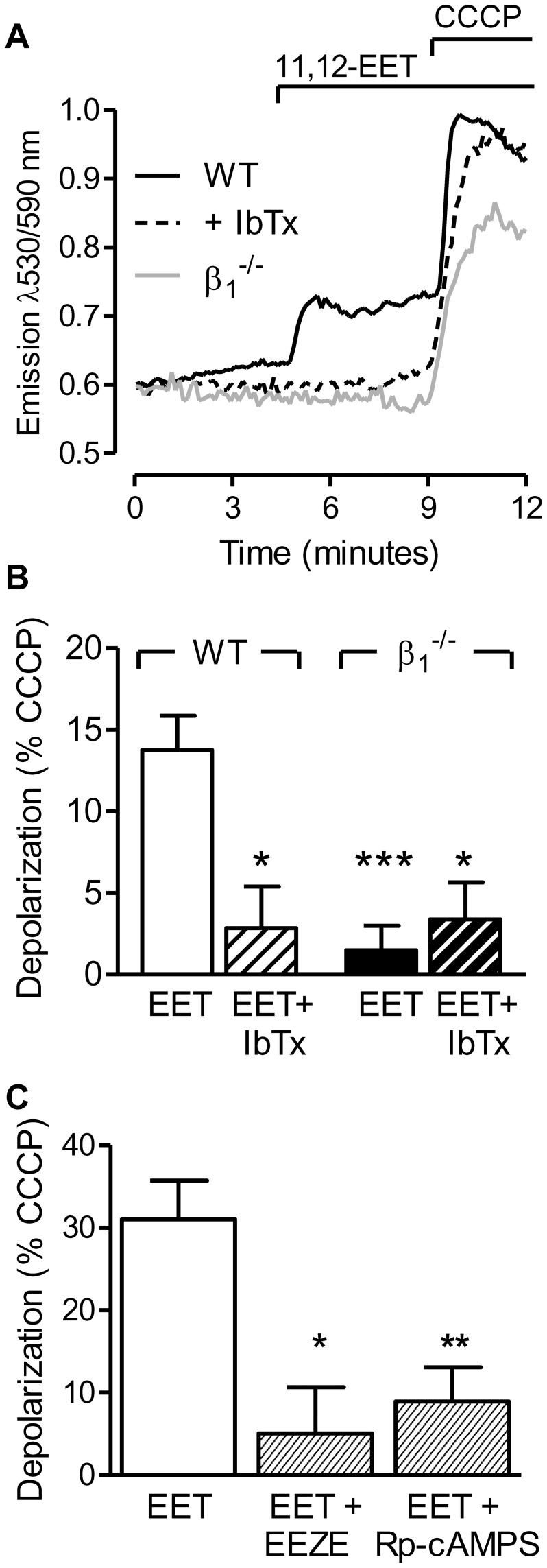
Effect of 11,12-EET on the mitochondrial membrane potential. (A) Representative tracing of the 11,12-EET (3 µmol/L)-induced changes in JC-1 fluorescence in pulmonary artery endothelial cells from wild-type (WT) cells in the absence or presence of iberiotoxin (IbTx, 300 nmol/L) and from BKβ_1_^−/−^ cells. (B) Mitochondrial membrane depolarization by 11,12-EET in WT and BKβ_1_^−/−^ pulmonary artery smooth muscle cells in the presence of solvent (Sol) or IbTx. (C) Mitochondrial membrane depolarization by 11,12-EET in WT cells in the presence of Sol, 14,15-EEZE (10 µmol/L), or Rp-cAMPS (10 µmol/L). All experiments were performed in the presence of diclofenac (10 µmol/L) and L-NA (300 µmol/L). The bar graphs summarize data obtained in 4–10 independent experiments; *P<0.05, ***P<0.001 versus WT+EET.

In pulmonary artery smooth muscle cells, a considerable portion of cellular BK channels are expressed in mitochondria [25], where opening is associated with a decrease in ΔΨ_m_ [26]. Indeed, the stimulation of JC-1-loaded, L-NA and diclofenac treated, cultured pulmonary artery smooth muscle cells with 11,12-EET (3 µmol/L) resulted in an increase in 530/590 nm fluorescence indicating a partial depolarization of the mitochondrial membrane. This effect was evident in cells from wild-type mice and could be inhibited with iberiotoxin but was not apparent in cells from BKβ1-deficient mice (Figure 5A&B).

EETs are thought to initiate their cellular responses by binding to a putative Gαs-coupled receptor and the downstream activation of protein kinase A (PKA) (for review see [10]). Therefore, we assessed the consequences of treating cells with the so-called EET antagonist 14,15-EEZE [15], and the protein kinase A inhibitor Rp-cAMPS on the loss in ΔΨ_m_ induced by 11,12-EET (Figure 5C). Addition of the direct BKα opener, NS1619 (1 µmol/L), depolarized the mitochondrial membrane in both cell types (85.5±5.5% and 88.7±11.7% of the depolarization to CCCP in wild-type and BKβ1^−/−^ cells, respectively). In isolated mitochondria from either WT or and BKβ1-deficient lungs, 11,12-EET failed to alter ΔΨ_m_ even though the BKα opener, NS1619 significantly reduced the membrane potential of isolated mitochondria from both strains (Figure S3). Taken together, these results suggest that BKα channels are present in the mitochondrial inner membrane of both cell types, but that 11,12-EET activates the channel via a receptor- and PKA-dependent signaling cascade.

### Effect of 11,12-EET on the Association of the BKα and BKβ1 Subunits

**Figure 6:**
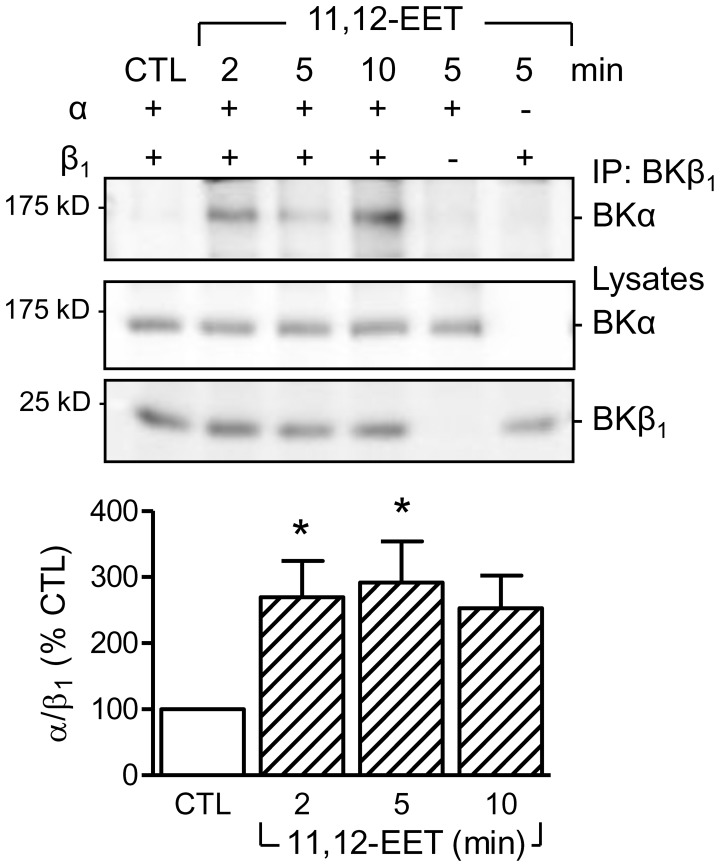
11,12-EET-induced association of the BKα and β_1_ subunits. Representative blot and densitometric analysis showing the co-precipitation of BKα with BKβ1 from HEK293 cells overexpressing either one or both BK subunits and stimulated with 11,12-EET (10 µmol/L) for 2–10 minutes. The graph summarizes data from 6 independent experiments; *P<0.05 versus the unstimulated control (CTL).

The β1 subunit sensitizes the BK channel to Ca^2+^ and voltage [27]. As the β1 subunit seemed to be essential to render the channel sensitive to EETs we determined whether or not 11,12-EET stimulated the association of the α and β subunits. To determine this, the α and β subunits of the BK channel were overexpressed in HEK293 cells and their subcellular localization studied. Using cell fractionation protocols it was possible to detect both channel subunits in the mitochondria (Figure S4). Immunoprecipitation of the β1 subunit revealed little or no association with the α subunit under basal conditions. However, the two proteins could be co-precipitated following cell stimulation with 11,12-EET. Subunit association was first assessed after 2 minutes and remained stable for at least 10 minutes (Figure 6).

## Discussion

The results of the present investigation indicate that in the absence of NO and PGI_2_, 11,12-EET is able to promote the association of the α and β1 subnunits of the BK channel in pulmonary artery smooth muscle cell mitochondria, to decrease the mitochondrial membrane potential and elicit vasoconstriction.

The functional pore of the BK channel is formed by four α subunits and conducts K^+^ currents in response to increases in Ca^2+^ and/or membrane depolarization, even in the absence of accessory subunits. The Ca^2+^ and voltage requirements for channel opening can be altered by the post-translational modification of the channel e.g. by phosphorylation or by its association with one of four potential β subunits. Of the four known β subunits, the β1 subunit is the best studied and its association enhances the voltage and Ca^2+^ sensitivity of the BKα [28], thus essentially increasing its open probability. EETs have long been linked with the activation of BK channels [10] but the molecular mechanisms involved have not been fully elucidated. The most likely explanation is that the fatty acid epoxides exert their cellular actions by binding to a specific cell surface receptor, thereby activating specific intracellular signaling cascades that effect channel opening. Certainly, specific EET binding sites have been detected on vascular smooth muscle cells [29] and many of the biological effects of the EETs, including the activation of overexpressed BKα subunits, are dependent on Gαs and the activation of PKA [30]. Current research is focused on identifying a specific EET-binding Gαs-protein-coupled receptor. Our finding that 11,12-EET PKA-dependently influenced mitochondrial membrane potential in intact cells but not in isolated mitochondria certainly supports this hypothesis. Moreover, the results of the present investigation reveal that the response to 11,12-EET is dependent on the β1 subunit. Indeed, we found that 11,12-EET promoted the association of the BKα and β subunits and were unable to detect a response to 11,12-EET in lungs or vascular smooth muscle cells from BKβ1^−/−^ mice.

There are several ways in which the EETs could target the β1 subunit. One possibility is by the EET-stimulated post-translational modification of the protein e.g. by phosphorylation. Certainly, many of the EET regioisomers are able to activate several intracellular kinases, including Src-family tyrosine kinases [31]. However, the known tyrosine phosphorylated residues tend to be within α-subunits and there is no known phosphorylatable regulatory site on the β1 subunit [32]. An alternative mechanism could be linked to EET-induced alterations in intracellular Ca^2+^ levels. In favor of this are a series of observations that link the EET-induced activation of BK channels with Ca^2+^ influx through TRPV4 channels in vascular smooth muscle cells [33], [34]. A similar phenomenon occurs in endothelial cells [35] and in pulmonary artery smooth muscle cells [8], in which the EET-stimulated Ca^2+^ influx and pulmonary vasoconstriction requires TRPC channel translocation to caveolae. A third possibility is that the EETs could directly influence the subcellular localization of the BK channel subunits and previous reports have linked the β1 subunit with the membrane localization of BKα [36]. Our data clearly demonstrate that 11,12-EET induced the association of BKα with β1 subunits and since this association markedly sensitizes the α-subunit to low levels of Ca^2+^ [37], it effectively increases open probability.

Activation of plasma membrane BK channels is generally associated with hyperpolarization and vasodilatation. Indeed, the BK channel opener NS1619 is reported to decrease pulmonary artery pressure in perfused rat lungs [38]. This fits with our own observations, as we observed a pronounced hyperpolarization of pulmonary artery smooth muscle cells stimulated with NS1619 in the presence of NOS and COX inhibitors. Nevertheless, stimulation of isolated cells or intact lungs with 11,12-EET induced an iberiotoxin-sensitive depolarization and increase in pulmonary artery pressure, respectively. These observations could imply that the EET-sensitive, β1-regulated BK channels in pulmonary artery smooth muscle cells form a small subpopulation either present at, or recruited to, a subcellular site where channel opening results in depolarization and constriction. Most studies on the BK channel have focused on plasma membrane hyperpolarization, however, it is also known to be present in the inner mitochondrial membrane [26], particularly in pulmonary artery smooth muscle cells [25]. The specific activation of mitochondrial BK channels could account for the EET-induced loss of the mitochondrial potential. Since in pulmonary smooth muscle cells mitochondria play a central role in buffering the cytosolic Ca^2+^ [3], and ΔΨ_m_ is a driving force for mitochondrial Ca^2+^ uptake [39], a reduction in ΔΨ_m_ through increased mitochondrial BK activity would be expected to enhance contractility.

We previously demonstrated that 11,12-EET and sEH inhibition were able to activate TRPC6 channels and the Rho kinase to increase pulmonary vascular tone in isolated lungs and pulmonary smooth muscle cells [8]. In the present study, we were unable to detect any role for the BK channel in the hypoxia- or 11,12-EET-induced pulmonary vasoconstriction unless NOS and COX enzymes were inhibited. Why was this the case? Initially, it was assumed that the presence of NO could attenuate EET production by inhibiting CYP epoxygenase activity. However, while this is certainly possible when high levels of NO are generated or in the presence of NO donors [40], many of the biological actions of the EETs e.g. on TRP channel activation [35], kinase activation [41], or on angiogenesis [42] do not seem to be impaired by basal levels of NO. One possible explanation is that in the presence of NO and PGI_2_, cyclic GMP- and cyclic AMP-dependent phosphorylation events activate BK channels at the plasma membrane [43], and this offsets the depolarizing effect of the EET-sensitive BK subpopulation in mitochondria. Nevertheless, we feel that the molecular mechanisms described here are of physiological/pathophysiological relevance as endothelial dysfunction and pulmonary hypertension are associated with impaired NOS activity/NO production [44] and PGI_2_ release [45], as well as increased EET production [6], reduced EET metabolism [12] and increased BKβ1 expression [46]. Combined, these alterations in the pulmonary vasculature would be expected to enhance the influence of EETs on the regulation of pulmonary artery tone.

## Supporting Information

Figure S1 **Effect of U46619 (1 nmol/L to 1 µmol/L) on pulmonary arterial pressure (ΔPAP) in lungs from WT and BKβ_1_^−/−^ mice in the presence of diclofenac (10 µmol/L) and L-NA (300 µmol/L).** The graph summarizes data obtained in 4 independent experiments.
(TIF)

Figure S2 **Fluorescent assessment of changes in the plasma membrane potential in Di-8-ANEPPS-loaded pulmonary artery smooth muscle cells.** (A) Effect of increasing concentrations of KCl in the presence of the K+ ionophore valinomycin (1.8 µmol/L). (B) The K+-induced changes in the emission ratio were plotted against the K+ potential calculated according to P = −61log[K+]i/[K+]o assuming [K+]i = 120 mmol/L, n = 5. (C) Effect of the BK channel opener, NS1619 (10 µmol/L), in the absence and presence of valinomycin and KCl. (D) The NS1619-induced hyperpolarization in pulmonary artery smooth muscle cells from wild-type (WT) and β1^−/−^ mice (n = 5–6).
(TIF)

Figure S3 **Membrane potential in isolated mitochondria.** (A) Expression of the BK channel in lung homogenates and purified mitochondria from wild-type (WT) and BKβ_1_^−/−^ mice. (B) Typical tracing and summary of the membrane depolarisation in WT pulmonary mitochondria induced by the solvent (DMSO 0.1%), 11,12-EET (10 µmol/L), or NS1619 (1 µmol/L); **P<0.01, n = 7–9. (C) Typical JC-1 fluorescence traces in WT and BKβ_1_^−/−^ lung mitochondria showing similar polarization and responses to NS1619 (1 µmol/L) and CCCP(0.1 µmol/L). The arrow head indicates the addition of mitochondria to the buffer. Identical responses were recorded in 3 additional experiments.
(TIF)

Figure S4 **Mitochondrial expression of BK α and β1 subunits in HEK293 cells.** HEK293 cells transfected with a control plasmid, the flag-tagged BKα channel or the β1 subunit. Total lysates and the mitochondrial fraction were isolated and probed for BK subunit expression. The voltage-dependent anion-selective channel 1 (VDAC1) was used as a mitochondrial marker.
(TIF)
