# Correction: 11,12-EET Stimulates the Association of BK Channel α and β_1_ Subunits in Mitochondria to Induce Pulmonary Vasoconstriction

**DOI:** 10.1371/annotation/d8c6c26e-1642-4c91-827c-f7f83d20be21

**Published:** 2012-12-07

**Authors:** Annemarieke E. Loot, Isabelle Moneke, Benjamin Keserü, Matthias Oelze, Tetyana Syzonenko, Andreas Daiber, Ingrid Fleming

The second and third authors, Isabelle Moneke and Benjamin Keserü should not have been attributed equal contribution to this work. The first and second authors, Annemarieke E. Loot and Isabelle Moneke, should be noted as contributing equally to this work. 

