# Supplementary figures and images for: 11,12-EET Stimulates the Association of BK Channel α and β1 Subunits in Mitochondria to Induce Pulmonary Vasoconstriction
Source: PLoS One. 2012 Sep 24;7(9):e46065. doi: 10.1371/journal.pone.0046065 (PMC3454360; doi:10.1371/journal.pone.0046065)

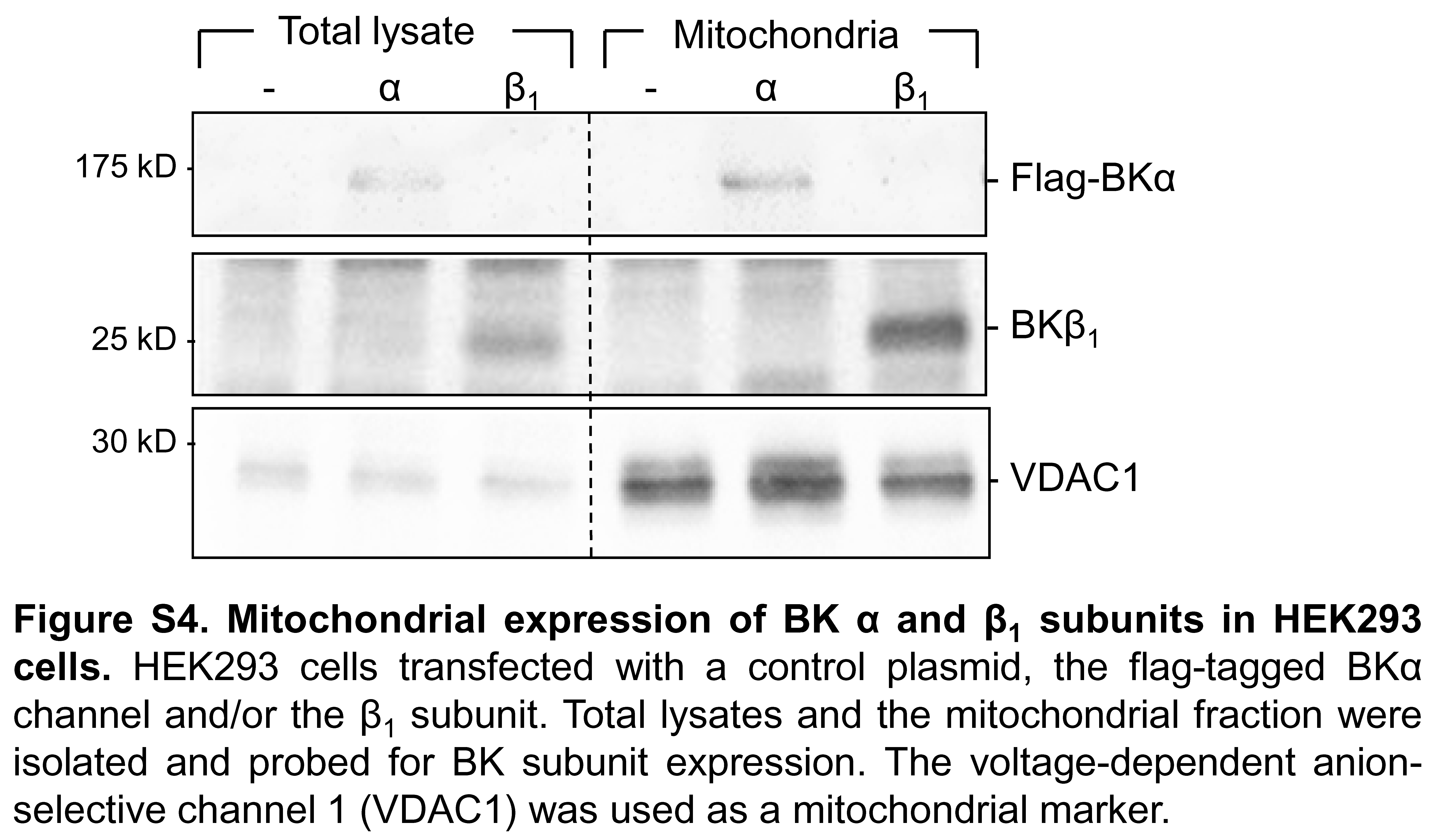

Supplement: Figure S4 — Mitochondrial expression of BK α and β1 subunits in HEK293 cells. HEK293 cells transfected with a control plasmid, the flag-tagged BKα channel or the β1 subunit. Total lysates and the mitochondrial fraction were isolated and probed for BK subunit expression. The voltage-dependent anion-selective channel 1 (VDAC1) was used as a mitochondrial marker. (TIF) [file pone.0046065.s004.tif]
